# Parallel Representation of Value-Based and Finite State-Based Strategies in the Ventral and Dorsal Striatum

**DOI:** 10.1371/journal.pcbi.1004540

**Published:** 2015-11-03

**Authors:** Makoto Ito, Kenji Doya

**Affiliations:** Okinawa Institute of Science and Technology Graduate University, Onna-son Okinawa, Japan; Indiana University, UNITED STATES

## Abstract

Previous theoretical studies of animal and human behavioral learning have focused on the dichotomy of the value-based strategy using action value functions to predict rewards and the model-based strategy using internal models to predict environmental states. However, animals and humans often take simple procedural behaviors, such as the “win-stay, lose-switch” strategy without explicit prediction of rewards or states. Here we consider another strategy, the finite state-based strategy, in which a subject selects an action depending on its discrete internal state and updates the state depending on the action chosen and the reward outcome. By analyzing choice behavior of rats in a free-choice task, we found that the finite state-based strategy fitted their behavioral choices more accurately than value-based and model-based strategies did. When fitted models were run autonomously with the same task, only the finite state-based strategy could reproduce the key feature of choice sequences. Analyses of neural activity recorded from the dorsolateral striatum (DLS), the dorsomedial striatum (DMS), and the ventral striatum (VS) identified significant fractions of neurons in all three subareas for which activities were correlated with individual states of the finite state-based strategy. The signal of internal states at the time of choice was found in DMS, and for clusters of states was found in VS. In addition, action values and state values of the value-based strategy were encoded in DMS and VS, respectively. These results suggest that both the value-based strategy and the finite state-based strategy are implemented in the striatum.

## Author Summary

The neural mechanism of decision-making, a cognitive process to select one action among multiple possibilities, is a fundamental issue in neuroscience. Previous studies have revealed the roles of the cerebral cortex and the basal ganglia in decision-making, by assuming that subjects take a value-based reinforcement learning strategy, in which the expected reward for each action candidate is updated. However, animals and humans often use simple procedural strategies, such as “win-stay, lose-switch.” In this study, we consider a finite state-based strategy, in which a subject acts depending on its discrete internal state and updates the state based on reward feedback. We found that the finite state-based strategy could reproduce the choice behavior of rats in a binary choice task with higher accuracy than the value-based strategy. Interestingly, neuronal activity in the striatum, a crucial brain region for reward-based learning, encoded information regarding both strategies. These results suggest that both the value-based strategy and the finite state-based strategy are implemented in the striatum.

## Introduction

Theoretical studies of decision-making have focused on the dichotomy of whether an environmental model is utilized, i.e. model-free or model-based strategies [1,2]. In a typical model-free strategy, called a value-based strategy, the goodness of each action candidate is memorized and learned directly from experienced sequences of state, action, and reward in the form of an action value function [2–5]. The hypothesis that such value-based strategies are implemented in the cortico-basal ganglia circuit[1,6] is supported by a growing number of reports of action-value coding neuronal activities in the striatum, the input site of the basal ganglia, in rats [5,7,8], monkeys [4,9–11], and humans [12]. By contrast, in a model-based strategy, the goodness of each action candidate is evaluated indirectly using an internal model of environmental state transitions. Recent fMRI studies found BOLD signals correlated with estimated states and state prediction errors in the prefrontal cortex [13–15].

While the value-based and model-based strategies have been helpful in dissecting the process of decision-making, the validity of such concepts and consequent predictions need to be assessed in light of actual animal and human behaviors. For example, animals often utilize a simple “win-stay, lose-switch” (WSLS) strategy, in which the same action is repeated if it is rewarded and switched if it is not rewarded [5,16]. This strategy does not conform to either the value-based or the model-based strategy. Theoretical studies have shown that optimal behavior under uncertain state observation can be represented as a finite state machine in which an action is selected depending on the agent’s discrete internal state, and the state is updated based on sensory observation and reward feedback [17]. The WSLS strategy is simply realized as a finite state machine with two states.

Here we consider the validity of the finite state-based strategy as another class of model-free strategy along with the value-based strategy in modeling animal choice behaviors. We reanalyze a part of the data we published previously [18], and we show that the finite state strategy fits the choice behavior of rats in a free-choice task more accurately than the value-based strategy and the model-based strategy. We further reanalyze the firing of phasically active neurons (PANs; putative medial spiny neurons) recorded from the dorsolateral striatum (DLS), dorsomedial striatum (DMS), and the ventral striatum (VS) during the task. We show that the individual states of the finite state strategy are encoded in DMS at the time of choice and that clusters of states are encoded in VS. Furthermore, the action values used in the value-based strategy are also encoded in DMS. These results suggest that both the value-based strategy and the finite state strategy are implemented in the striatum.

## Results

**Fig 1:**
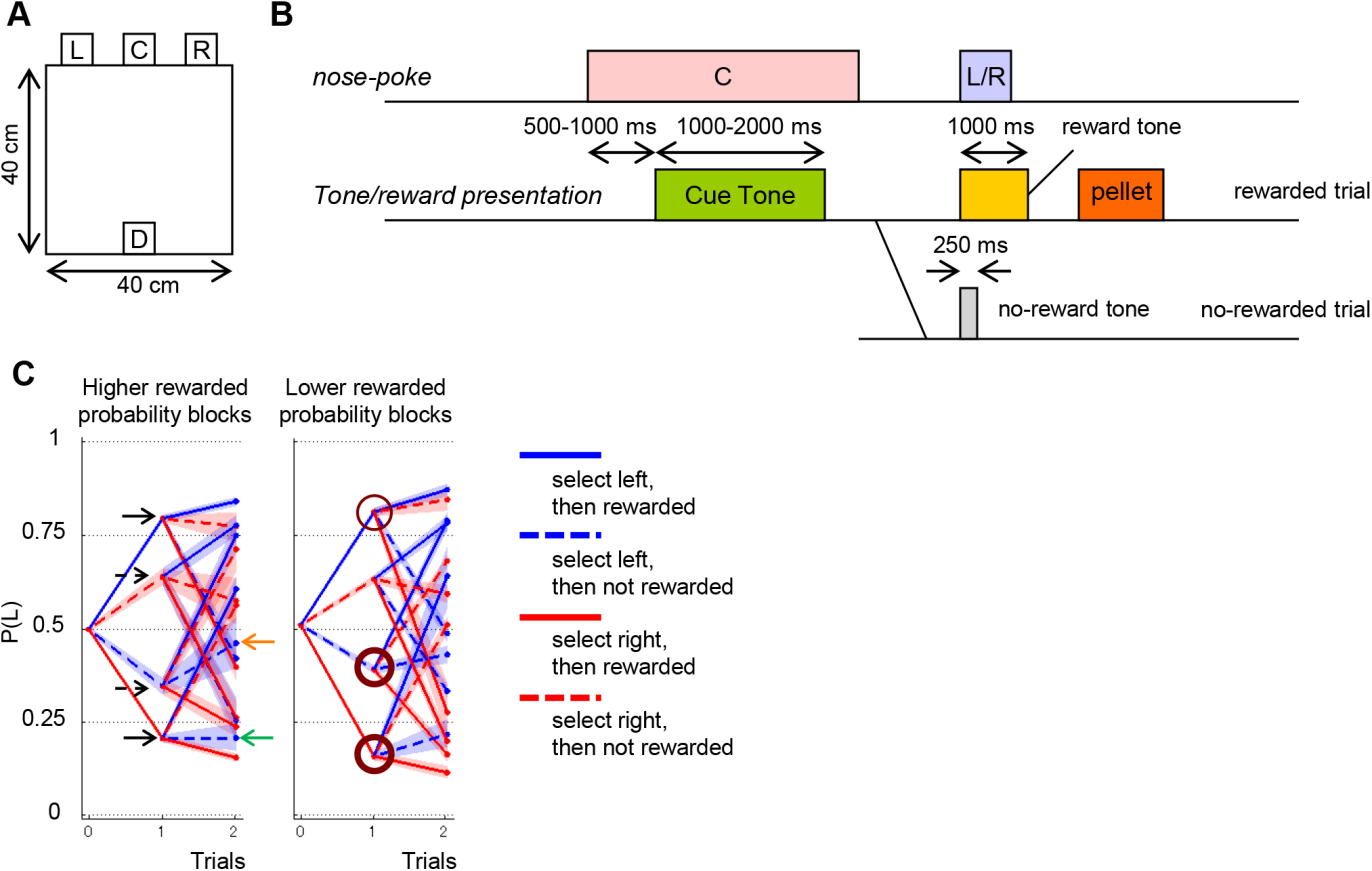
Design of the choice task. (A) A schematic illustration of the experimental chamber. The chamber was equipped with three holes for nose poking (L, left hole; C, center hole; R, right hole) and a pellet dish (D).(B) The time sequence of the choice task. When a rat performed a nose poke in the center hole for 500–1,000 ms, a cue tone (white noise) was presented. The rat had to maintain the nose poke during the presentation of the cue tone, or the trial was terminated as an error trial after presentation of an error tone. After the cue tone, the rat was required to perform a nose-poke in either the left or right hole. Then either a reward tone or a no-reward tone was presented stochastically depending on the rat’s choice and the current left-right probability block. The reward tone was followed by delivery of a sucrose pellet to the pellet dish. Reward probabilities for left and right nose pokes were selected from four pairs [(left, right), (90%, 50%), (50%, 90%), (50%, 10%), and (10%, 50%)]. The probability pair was fixed during a block. Subsequently, the reward probability setting was changed when the choice frequency of the more advantageous side during the last 20 choice trials reached 80%. For this calculation, the same block was held until at least 20 choice trials were completed. A session consisted of four blocks, and the sequence of the reward probability pairs was given in a pseudorandom order, so all four pairs were used once per session. (C) Decision trees averaged by all rats. The left choice probability for all possible experiences in one and two previous trials in the higher reward probability blocks (left) and in the lower reward probability blocks (right). Four types of experiences in one trial [left or right times rewarded (1) or no reward (0)] are represented by different colors and line types. For instance, left probability after L1, *P*(L|L1), is indicated by the right edge of a blue solid line (upper black solid arrow in the left panel), and left probability after R1 L0 (R1 and then L0), *P*(L|R1 L0), is indicated by the right edge of a blue broken line connected to the red solid line (green arrow). Values of trials = 0 (x-axis) represent the left choice probability for all trials. Shaded bands indicate 95% confidence intervals. Significant differences in left choice probabilities for one previous trial between the higher and lower reward probability blocks are marked by brown circles in the right panel (thick circles for *p* < 0.01, a thin circle for *p* < 0.05; chi-squared tests).

In our previous study [18], we gathered behavioral and neuronal data during free-choice and forced-choice tasks. In the present study, we reanalyzed the dataset from free-choice tasks (Fig 1A and 1B), where rats were required to perform a nosepoke to either the left or right hole after cue-tone presentation. A food pellet was delivered probabilistically depending on the selected action. Reward probabilities were varied in a block-wise manner. The dataset contained behavioral and neuronal data from 34,459 trials (202 sessions) involving seven male Long-Evans rats (250–350 g body weight). Neuronal data comprised spike-timing of phasically activity neurons (PANs; putative medium spiny neurons): 204 PANs from the dorsolateral striatum (DLS), 112 PANs from the dorsomedial striatum (DMS), and 138 PANs from the ventral striatum (VS).

All rats successfully adapted to changing reward probabilities. The number of trials needed to reach the block change criterion was smaller in the higher reward probability blocks ((90%, 50%) and (50%, 90%); 33.9 trials on average with a standard deviation of 23.9 trials) than in the lower reward probability blocks ((50%, 10%) and (10%, 50%); 48.9 trials on average with a standard deviation of 29.4 trials, Mann-Whitney U test, *p* < 0.0001). These numbers are significantly smaller than the number required for random choices to reach 80% optimal by chance (about 713 trials; estimated by Monte Carlo method).

We first analyzed how rat choices depended on past experience by calculating decision trees [5,18] in the higher and lower reward probability blocks (Fig 1C). There are four possible types of experience in each trial: L1, L0, R1, and R0, where L or R denotes left or right choice, respectively, and 1 or 0 denotes rewarded or non-rewarded trials, respectively. Averaging all rats, left choice probability after L1, *P*(L|L1) was higher than 0.5 and its symmetric case, *P*(L|R1), was lower than 0.5 (namely, *P*(R|R1) = 1—*P*(L|R1) was higher than 0.5), indicating that a rewarded experience reinforced the tendency for the same choice in the next trial (black solid arrows, Fig 1C). On the other hand, left choice probability after L0, *P*(L|L0) was less than 0.5 and its symmetric case, *P*(L|R0), was larger than 0.5 (namely, *P*(R|R0) was less than 0.5), indicating that a non-rewarded experience increased the tendency to choose a different action in the next trial (broken arrows, Fig 1C). For all rats, staying tendency was stronger than switching tendency.

Not only the experience of the previous trial, but also the experiences before the previous trial affected choices. There are 4 x 4 = 16 possible experiences in two consecutive trials, and the left choice probability after each experience is plotted at trial 2 in Fig 1C. For instance, *P*(L|R1 L0), the left choice probability at trial *t* after R1 at trial *t—*2 then L0 at trial *t—*1 (green arrow, Fig 1C) is less than *P*(L|L0 L0), the left choice probability after double L0 experiences (orange arrow, Fig 1C), even though the experiences in the previous trial, L0, are the same.

The decision tree was affected by the reward probability setting (Fig 1C). The staying tendency was significantly stronger in the lower reward probability blocks than in the higher reward probability blocks (*p* < 0.05 for L1, *p* < 0.01 for R1, chi-squared test), and the switching tendency following unrewarded left choice (L0) was significantly stronger in the higher reward probability blocks than in the lower reward probability blocks (*p* < 0.01 for L0, *p* = 0.62 for R0, chi-squared test).

### Model-fitting to rat choice behavior

Next we explore more detailed descriptions of choice behavior using computational models that can predict rat choices based upon past experiences. Along with the Markov models and the value-based strategy tested in our previous study [18], we tested the model-based strategy and the finite state strategy.

#### Model-based strategy

For the model-based strategy, we introduced the environmental state estimate (ESE) model, which estimates the current reward setting from past experience using the knowledge that reward probabilities should be one of the following: (Left, Right) = (90%, 50%), (50%, 10%), (50%, 90%), and (10%, 50%). The estimated reward setting is used to calculate action values for left and right, which determine action probability. The performance of this model is characterized by two parameters; the block transition probability *ε*, and the magnitude of reward *κ*. The previous study showed that a binary version of the ESE model could explain human choice behavior better than reinforcement learning models [13]. As in the Q-learning with differential forgetting (DFQ-learning model) [18], we considered cases of fixed and time-varying parameters (for more detail, see Materials and Methods).

#### Finite state-based strategy

As a computational model for a finite state-based strategy, we introduced the finite state agent (FSA) model, which assumes that an animal has an internal state variable *x* that can assume *N* possible states from 1 to *N*. An action is selected stochastically according to the action probability associated with each state. After execution of an action and feedback of the reward outcome, the state changes according to state transition probabilities. Free parameters of the FSA model are the initial distribution of states, the action probability distribution at each state, and the state transition probability matrix for each action and reward outcome. The FSA model can be regarded as an extended version of the hidden Markov model (HMM). However, unlike the HMM, in the FSA model, the state transition probability depends on the selected action and the reward outcome. In the HMM, the Baum-Welch algorithm [19], a form of the expectation-maximization (EM) algorithm, is used to find parameters that maximize the likelihood of the given data. We reformulated the Baum-Welch algorithm for the FSA model (see Materials and Methods) to fit its parameters to action and reward sequence data. Note that the FSA model is a descriptive model. It can mimic a choice behavior of an animal, but does not explain how the behavior is acquired by the animal.

#### Evaluation of models

**Fig 2:**
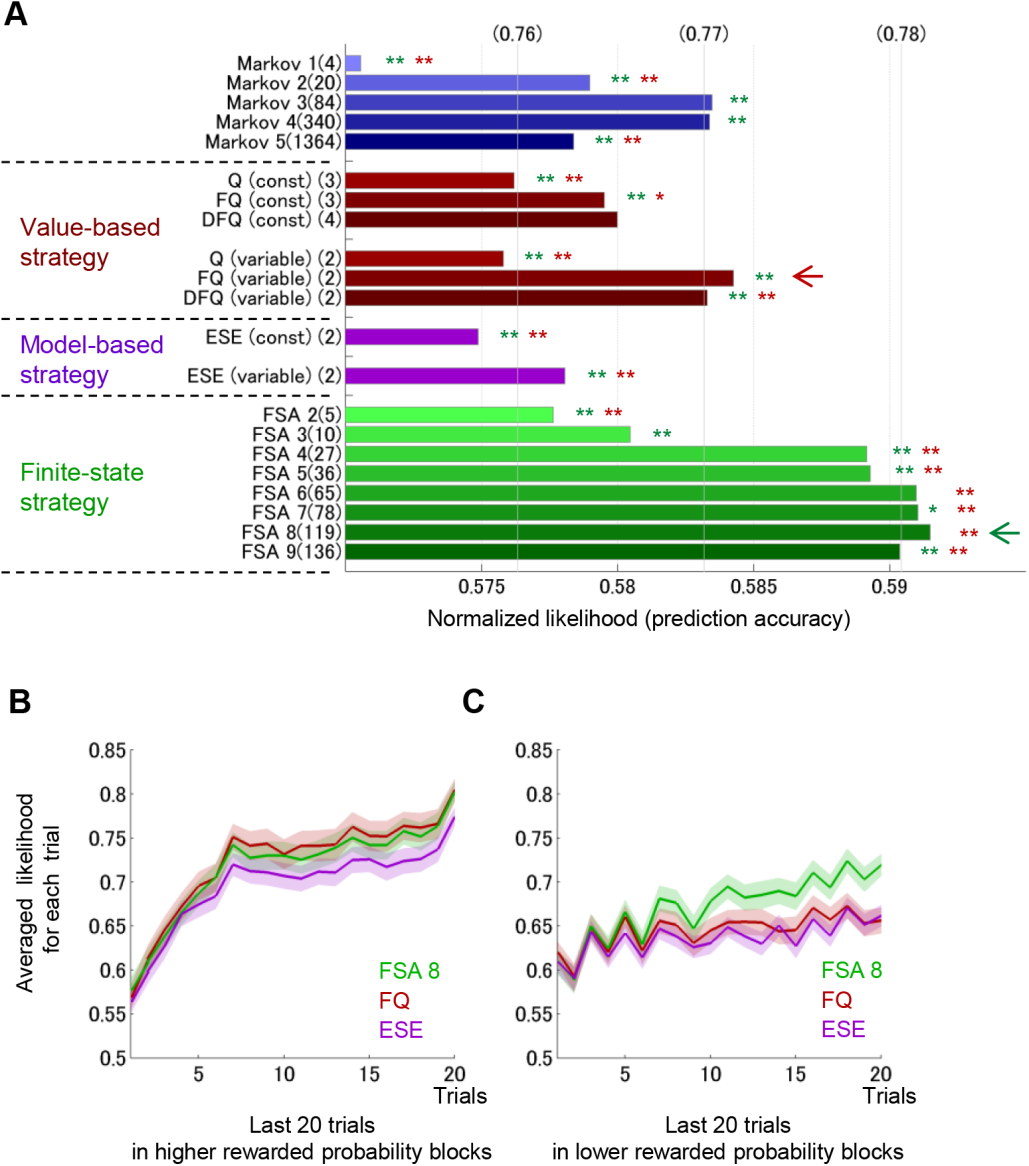
Comparison of model fits. (A) Normalized likelihoods for the Finite-state and Model-based strategies. For comparison, published data in Ito & Doya 2015, the likelihoods of Markov models and Value-based strategy are also shown. The fitness of the models was measured by the normalized likelihood of the test data, which were obtained from the geometric average of prediction accuracy for unknown data. Numbers in parentheses on the upper x-axis correspond to arithmetic averages of prediction accuracy. Numbers followed by the name of the model indicate numbers of free parameters in each model. “const” or “variable” means that the parameters of each model were assumed to be constant or variable, respectively. Green and brown asterisks indicate a significant difference from the normalized likelihood of the FSA model with 8 states (green arrow) and the FQ-learning model with variable parameters (brown arrow), respectively. ** for *p* < 0.01 and * for *p* < 0.05 in a paired-sample Wilcoxon test (See Materials and Methods). (B, C) Averaged likelihoods and standard errors (shaded bands) in last 20 trials in the higher (B) and the lower (C) reward probability blocks for the FQ-learning model with variable parameters (red), the FSA with 8 states (green), and in the ESE model with variable parameters (purple).

To evaluate how well the FSA and other models predict rat behaviors, we divided the behavioral data into training data (17603 trials, 101 sessions) and test data (16856 trials, 101 sessions). Free parameters of the models were determined to maximize the likelihood of the training data. We compared the performance of the models by the normalized likelihood of the test data, which shows the prediction accuracy of the choice data not used for parameter search (Fig 2A; see Materials and Methods).

The performance of the 3rd-order Markov models was the highest in the Markov models, and decreased in 4th- and 5th-order models due to over-fitting [18]. Within the value-based strategy, the Q-learning with forgetting (FQ-learning) with time-varying parameters showed the highest performance, exceeding that of the best Markov model [18]. The performance of the model-based strategy (ESE models) was less than that of the 2nd-order Markov model and significantly less than the best Q-learning model, suggesting that rats probably do not use the model-based strategy in this choice task (The parameters of the Q-learning models and the ESE models are reported in Tables 1 and 2).

**Table 1:** Summary of free parameters of Q-learning models.

	# of parameters	*α*_1_	*α*_2_	*κ*_1_	*κ*_2_	*σ*_*α*_	*σ*_*κ*_
**standard Q (const)**	3	0.50		1.9	0.4		
**F-Q (const)**	3	0.50	*α*_1_	2.1	1.0		
**DF-Q (const)**	4	0.50	0.20	2.0	0.7		
**Standard Q (variable)**	2					0.10	0.13
**F-Q (variable)**	2					0.12	0.09
**DF-Q (variable)**	2					0.11	0.06

**Table 2:** Summary of free parameters of ESE models.

	# of parameters	*ε*	*κ*	*σ*_*ε*_	*σ*_*κ*_
**ESE (const)**	2	0.19	6.9		
**ESE (variable)**	2			0.02	0.26

For the FSA models, interestingly, even with 4 internal states, the likelihood surpassed that of the best reinforcement learning model, the FQ-learning model with varying parameters. The likelihood of the FSA models increased as the number of states increased, with a peak at *N* = 8. The likelihood of the FSA model with 8 states was significantly higher than all other models (one-sided Mann-Whitney U test, *p* < 0.05) except for the FSA model with 6 states. With *N* = 9 or more states, likelihood decreased due to over-fitting.

To clarify why the FSA model performed better than other models, we compared the average likelihood of the best models from the three strategies in the last 20 trials in higher reward probability blocks (Fig 2B) and lower reward probability blocks (Fig 2C). In the later part of higher reward probability blocks, the FSA model and the FQ model showed higher likelihoods than the ESE model. In the later part of lower reward probability blocks, the FSA model showed much higher likelihood than the other two models. The averaged likelihood had an increasing tendency throughout the trials in a block. Because each block ended when the choice probability of the more advantageous side reached 80%, the tendency of selecting the optimal side was stronger in the later part of the block. Therefore, it was easier for models to predict actions in the later. This is the reason for the increasing tendency of the averaged likelihood. As a result, differences between models were clearer in the later part of the block.

**Fig 3:**
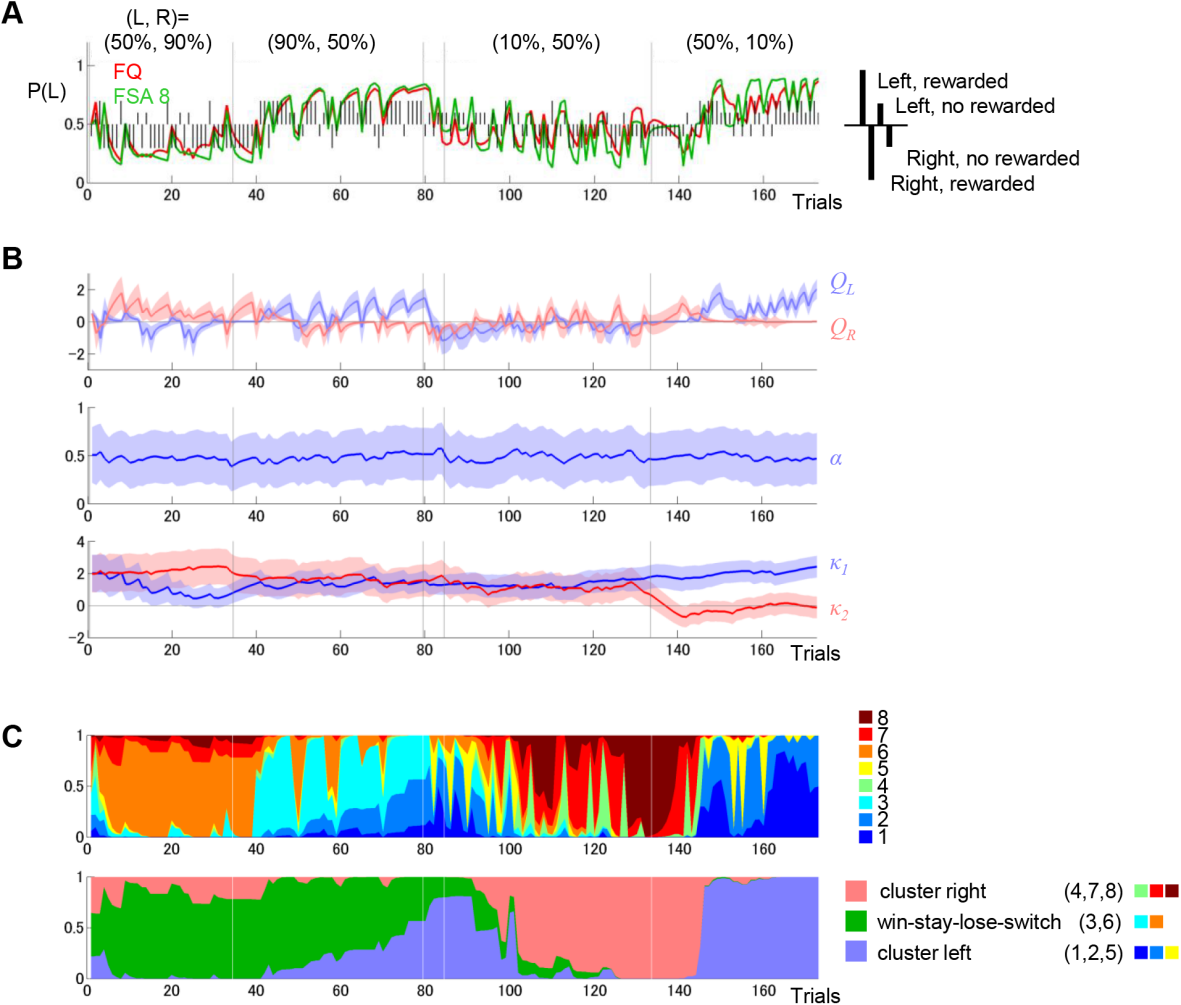
An example of behavioral performance and model fits. (A) An example of behavioral performance and predictions made by the models. Vertical black lines indicate rat choice behavior. Left and right choices are represented by upper and lower bars, respectively. Rewarded and non-rewarded outcomes are represented by long and short bars, respectively. Model fits, representing the prediction probability that the rat selects left at trial *t*, were estimated using previous choices and the reward outcomes from trial 1 to *t*-1 based on the FQ-learning or the FSA model with 8 states. These are represented by red or green lines, respectively. (B) Estimated action values and varying parameters of the FQ-learning model and standard deviations of posterior probabilities. *Q*_*L*_ and *Q*_*R*_, action values for left and right; *α*, the learning rate for the selected action (= forgetting rate for the action not chosen); *κ*_1_, the strength of reinforcement by reward; and *κ*_2_, the strength of the aversion resulting from the no-reward outcome. (C) Posterior probabilities of internal states (upper panel) and clusters (lower panel) of the FSA model with 8 states shown by stacked graphs. The index of states and clusters corresponds to the index in Fig 4C.

Higher likelihood is obtained by a correct prediction with higher confidence (Eq (4) in Materials and Methods). For example, in the trials that the rat selected the left hole, the prediction *P*_*L*_ = 0.8 (*P*_*L*_: the predictive probability that the rat would select left) results in higher likelihood than the prediction *P*_*L*_ = 0.7. In the trials that the rat selected right, the prediction *P*_*L*_ = 0.2 results in higher likelihood than the prediction *P*_*L*_ = 0.3. It is consistent with the predictive mode of the FSA model, showing more pronounced changes in action choice probability in lower reward blocks than the FQ model (Fig 3).

**Fig 4:**
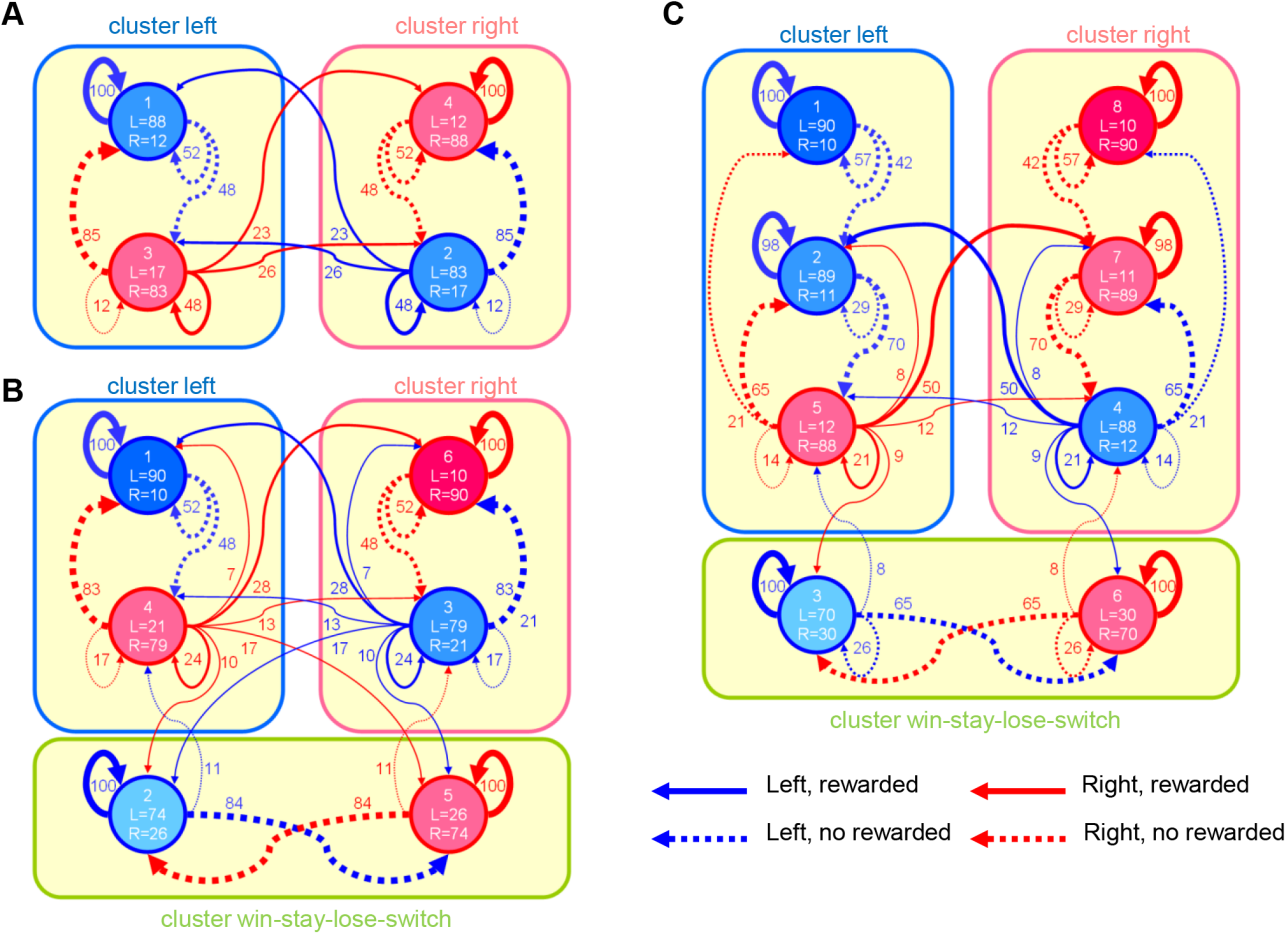
Estimated parameters of finite state agent (FSA) models. Each state is represented by a blue or red circle, and numbers in circles represent indices of state and action probabilities (%) for left and right. States for which the probability of left (right) is larger than that of right (left) are shown in blue (red). Each arrow with a number indicates the transition probability (%) after left (blue) or right (red) is chosen and a reward is obtained (solid) or not obtained (dashed). For simplicity, only transition probabilities greater than 5% are shown. These parameters were estimated under symmetric constraints. States form clusters that represent different sub-strategies (cluster left, cluster right, and win-stay, lose-switch). See Materials and Methods for the mathematical definition of the clusters. (A) FSA model with 4 states, (B) 6 states, and (C) 8 states.

With *N* = 4 (Fig 4A), the four states formed two clusters (states 1 and 3, and states 2 and 4) corresponding to the sub-strategies or the belief that “left is better” (cluster left) and “right is better” (cluster right), respectively. In state 1, the model selects left with a high probability (88%) and stays there if it is rewarded, but moves to state 3 with a 48% probability if it is not rewarded. In state 3, the model can be interpreted as doubting the current belief that left is better; the model tries right (83%) and returns to state 1 if it is not rewarded (doubt is cleared), but transits to state 2 or 4 with a 49% probability if it is rewarded (doubt is confirmed). The transition probability for both beliefs is symmetric because we applied a symmetric constraint for the parameters (see Materials and Methods). We also tested the FSA models without a symmetric constraint, but performance was worse than with the constraint. When the number of states increased to *N* = 6, the model has an additional cluster composed of states 2 and 5 (cluster win-stay, lose-switch) (Fig 4B). In this cluster, as long as the reward is obtained, the same action is selected. Otherwise, the state is changed with an 84% probability, and the model switches the action.

The best FSA model with *N* = 8 states has additional states, 2 and 7, in left and right clusters, respectively (Fig 4C). These additional states allow the model to represent the degree of beliefs more finely. The model believes “left is better” more strongly in state 1 than in state 2, which can directly transit to state 5, where the model doubts the current belief. In the example shown in Fig 3A and 3C, the estimated internal states were mostly in the win-stay, lose-switch cluster during higher reward probability blocks (50%–90% and 90%–50% for left-right) and in the left or right cluster during lower reward probability blocks (10%–50% and 50%–10% for left-right). It is consistent with the property of the win-stay, lose-switch strategy, which is effective only when the reward probability for the optimal action is high.

### Comparison of simulated model behaviors with actual rat behaviors

While the likelihood of a model fitted to given choice sequences is a useful criterion for comparing models, it is also important to check how the model performs when it runs autonomously. One direct way to check this performance is to compare statistical features of the behavioral sequences produced by the model in a simulation with performance of rats in the actual task (see Materials and Methods). We simulated the Q, FQ, DFQ, and ESE models with constant parameters and the FSA models with 4, 6, and 8 states. We excluded the models with variable parameters because the random walk assumption was effective for fitting a model to a given choice sequence, but not for the generation of choice sequence in a free run.

**Fig 5:**
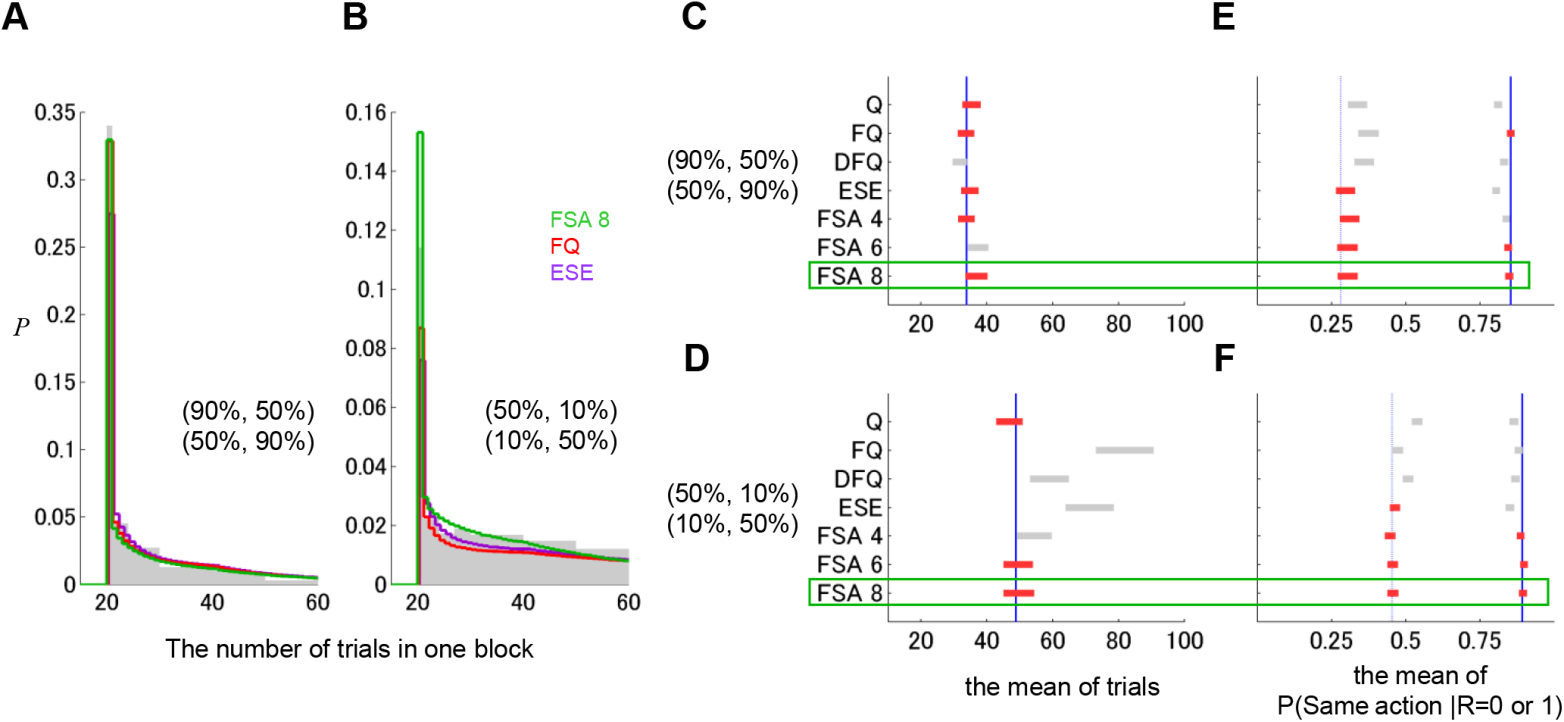
Comparison of simulated model behaviors with actual rat behaviors. (A, B) Distributions of trials needed to reach the 80% optimality criterion for rats (gray), FSA with 8 states (green), FQ with constant parameters (red), and ESE with constant parameters (purple) for blocks with higher reward probabilities (A) and for blocks with lower reward probabilities (B). (C, D) The mean number of trials in one block. Data from rats are indicated by blue vertical lines, and confidence intervals (100-5/6%; Bonferroni Method) of the hypothesis that the behavioral data were replicated by each model are represented by horizontal lines. The red color of confidence intervals means that behavioral data are within the confidence interval. (E, F) The mean probability that the same action is selected after rewarded (solid blue lines) and non-rewarded (dashed blue lines) trials, and corresponding confidence intervals of the models (horizontal lines).

We took the number of trials required to reach the block-change criterion (80% or more optimal choices in the last 20 trials) as a measure of the flexibility of adaptation (Fig 5A–5D) and the probability that the same action was selected after the rewarded or non-rewarded trial, *P*(*a*(*t*+1) = *a*(*t*)| *r*(*t*) = 1) and *P*(*a*(*t*+1) = a(*t*)| *r*(*t*) = 0), respectively, as a measures of the robustness of the action (Fig 5E and 5F). Statistics were calculated separately for blocks with higher reward probability settings [(90, 50%) and (50, 90%)] and lower reward probability settings [(50, 10%) and (10, 50%)].

We tested the hypothesis that data from rats could be generated from each model using the mean of the six statistics (Fig 5C–5F). Only the FSA model with 8 states was not rejected by any statistical test (the level of the confidence interval for each statistic was set to (100–5/6)%, so that the chance of at least one false rejection is 5%; Bonferroni Method). This result shows that only the FSA model with 8 states sufficiently reproduces the behavior observed in the rats, although it does not exclude the possibility that there are other models better than the FSA model with 8 states.

### Neural coding

Previous studies have shown that striatal neurons code not only observable behavioral variables, such as action and reward [5,7,10,20–22], but also hidden variables estimated from behavior using computational models, such as action values [4,5,7,12,23]. In our previous study [18], regression analysis revealed that action values, which were estimated from behavioral data based on the FQ-learning with variable parameters, were coded most strongly in DMS during action execution.

In this analysis, we re-analyzed the same neuronal data to examine whether a new class of hidden variables, namely, states and state clusters of the FSA with 8 states, were also coded. However, if we use a regression model that employs only states and clusters as regressors, it would lead to Type I errors (false positives). For instance, the estimate of state 1 is strongly correlated with the left action choice in the same trial, detecting action-coding neurons as state-coding neurons (Fig 4C). To avoid this problem, we first considered a full model including all possible variables (30 variables) that might be coded by striatal neurons (Poisson regression model, see Materials and Methods). Then, we extracted only the important variables to explain the output using lasso regularization [24] (see Materials and Methods). The full model we used was:

$$\begin{array}{l} \log\mu (t)=\beta_{0}+\beta_{b}b(t)+\beta_{a}a(t)+\beta_{r}r(t)+\beta_{a'}a(t-1)+\beta_{r'}r(t-1)\\ \;+\beta_{QL}Q_{L}(t)+\beta_{QR}Q_{R}(t)+\beta_{QC}Q_{c}(t)+\beta_{V}V(t)+\beta_{PLQ}P_{L:Q}(t)\\ \;+\beta_{x1}x_{1}(t)+\beta_{x2}x_{2}(t)+\cdots +\beta_{x8}x_{8}(t)\\ \;+\beta_{x1'}x_{1}(t+1)+\beta_{x2'}x_{2}(t+1)+\cdots +\beta_{x8'}x_{8}(t+1)\\ \;+\beta_{CL}C_{L}(t)+\beta_{CR}C_{R}(t)+\beta_{CW}C_{WSLS}(t)+\beta_{PL:FSA}C_{L:FSA}(t) \end{array}$$ (1)

where *μ*(*t*) is the expected number of spikes at trial *t* in a certain time bin and *β*_*i*_ is the regression coefficient for each explanatory variable (regressor). *b*(*t*) is the monotonically increasing factor, namely, *b*(*t*) = *t*, which is inserted to capture the task event-independent monotonic increases or decreases in firing pattern. The remaining regressors are classified into three types:

1. observable information: *a*(*t*) ∈ {1: Left, 2: Right}, the selected action; *r*(*t*) ∈ {1: Rewarded, 0: Non-rewarded}, reward availability; and *a*(*t*-1), and *r*(*t*-1), action and reward in the previous trial, respectively.
2. estimated information based on the FQ-learning: *Q*_*L*_(*t*) and *Q*_*R*_(*t*), action values estimated by the FQ-learning model with varying parameters [4,5,7,9,11,12,23]; *Q*_*c*_(*t*) ≡ *Q*_*a*(*t*)_(*t*), the action value for the selected action (chosen value) [7,23,25]; *V*(*t*) ≡ *P*_*L*:*Q*_(*t*)*Q*_*L*_(*t*) + (1 – *P*_*L*:*Q*_(*t*))*Q*_*R*_(*t*), the state value as defined by the average of action values [5,25]; and *P*_*L*:*Q*_(*t*), the action probability estimated by the FQ-learning model
3. estimated information based on the FSA model: *x*_1_(*t*),…, *x*_8_(*t*) (same as *γ* ^*n*^(*t*) in the FSA model, see Materials and Methods), the posterior probabilities of states that the FSA with 8 states may take at trial *t*; *x*_1_(*t*+1),…, *x*_8_(*t*+1), the posterior probabilities of transited states; *C*_*L*_(*t*), *C*_*R*_(*t*), and *C*_*WSLS*_(*t*), the posterior probabilities of clusters, namely, the sum of the corresponding state probabilities; and *P*_*L*:*FSA*_(*t*), the action probability estimated by the FSA model.

**Fig 6:**
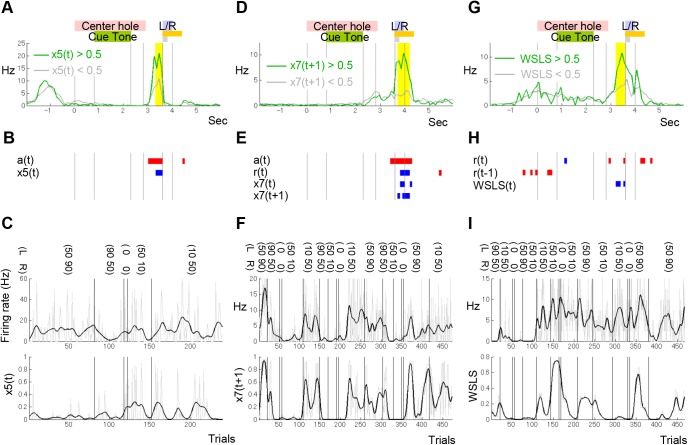
Examples of neuronal activities correlated with internal variables in the FSA model with 8 states. (A) Firing activity of a DMS neuron that was correlated with the posterior probability of state 5 of the FSA model with 8 states. Firing rates in trials where the estimated posterior probability of state 5 was high and low were shown by green or gray event-aligned spike histograms (EASHs; see Materials and Methods). (B) Information coded in the neuron shown in (A). Blue and red time bins for each regressor indicate time bins where neuronal activity was positively and negatively correlated with the regressor, respectively. In regressors selected by lasso from the 30 regressors for each time bin, only regressors that were detected for more than two adjacent time bins are shown (two regressors, in this case). (C) The correlation between firing rate and posterior probability of state 5. The firing rate in yellow time bins shown in (A) and the posterior probability of state 5 for each trial are plotted with gray lines in the upper and lower panels, respectively. Black lines were smoothed with a Gaussian filter using the standard deviation of three trials. (D, E, F) Firing activity of a DMS neuron that was correlated with state 7 at the next trial estimated by the FSA model with 8 states. (G, H, I) Firing activity of a VS neuron that was correlated with the sub-strategy (win-stay, lose-switch; WSLS) estimated by the FSA model with 8 states.

We applied lasso to this full model, which can identify minimally important regressors among many and redundant regressors (see Materials and Methods). When lasso identified certain regressors to explain the activity of a certain neuron, we interpreted this to mean that “the neuron coded the regressors.” A single striatum neuron tended to code multiple variables in different time bins as shown in Fig 6. Lasso detected significant populations of neurons that coded observable information (I) and estimated information based on the FQ-learning (II), similar to our previous analysis [18]. In addition, this analysis detected neurons that coded states of the FSA model (III). Fig 6A–6C show an example of DMS neurons in which firing rate was significantly correlated with the posterior probability of states of the FSA model. During action selection, firing rate was best explained by the regression model including not only the action, but also *x*_5_(*t*), in which the FSA model doubts the current belief that left hole is better and wants to choose the right hole (see Fig 4C). Fig 6D–6F show an example of DMS neurons in which firing rate was significantly correlated with the posterior probability of a transited state of the FSA model. The firing rate during the rat’s entry to the left or right hole (note that the reward or non-reward tone was presented at the onset of the hole poke) was best explained by the regression model, including not only the action, reward, and *x*_7_(*t*), but also *x*_7_(*t*+1). Here the FSA model believes the right hole is better following an exploratory choice (see Fig 4C). Fig 6G–6I show an example of VS neurons coding the rat’s sub-strategy (cluster). There was a significant, positive correlation between neuronal firing rate during action selection and the posterior probability of the win-stay, lose-switch cluster estimated by the FSA model with 8 states.

**Fig 7:**
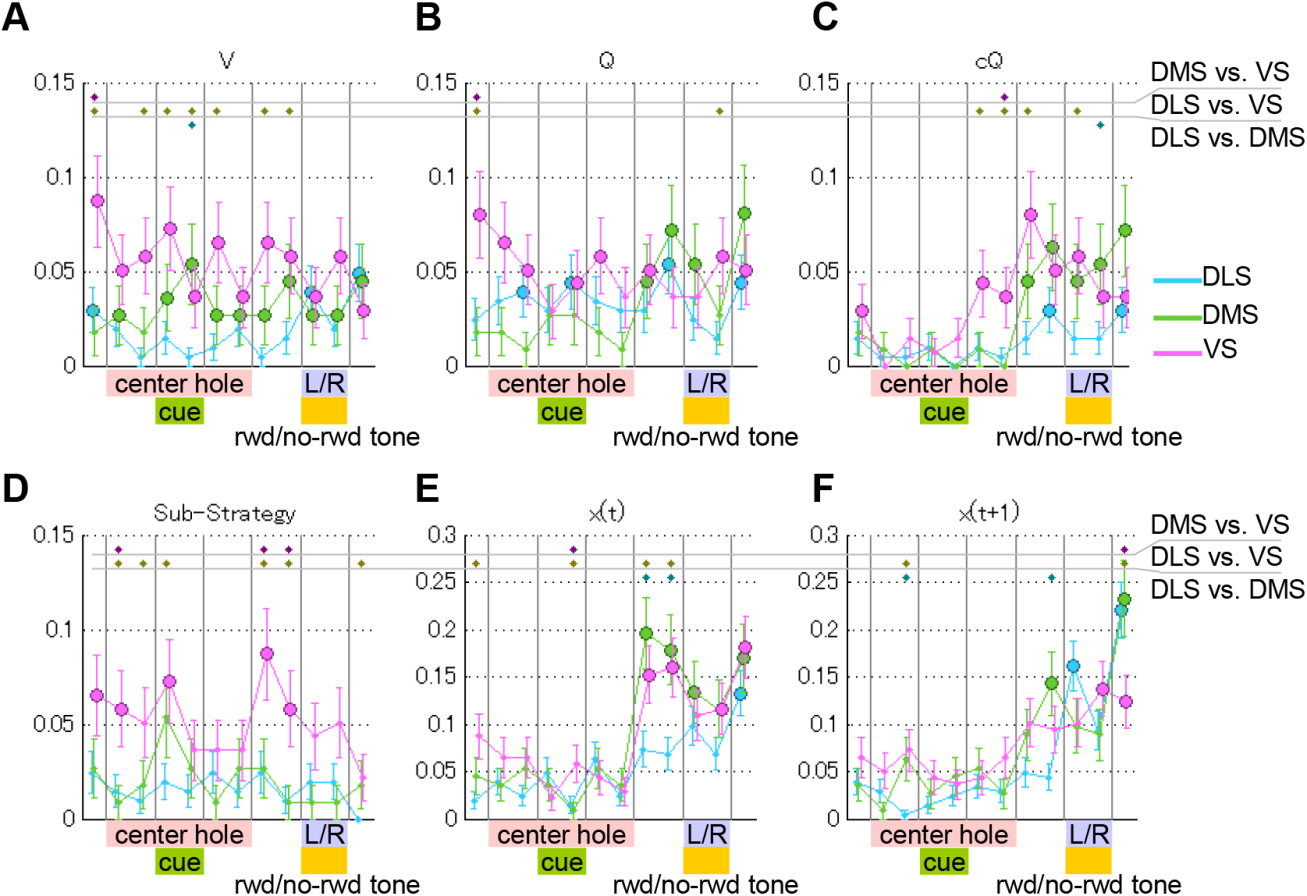
Proportions of neurons coding variables of the value-based and finite state-based strategies. Proportions of neurons showing significant correlations (*p* < 0.01, t test) with variables of the value-based strategy (FQ-learning) (A, B, C) and the finite state-based strategy (FSA model with 8 states) (D, E, F). These neurons were detected by lasso regularization of a Poisson regression model, which was conducted for 500 ms before and after the seven trial events (entry into the center hole, the tone onset, the tone offset, the exit from the center hole, the entry into the L/R hole, and the exit from the L/R hole) for DLS (blue), DMS (green), and VS (pink). Colored disks mean that the populations are significantly higher than by chance (*p* < 0.05, binominal test). (A) Neurons coding state values, the average of action values. (B) Neurons coding action values, *Q*_L_ and/or *Q*_R_. (C) Neurons coding chosen values, action values for the selected action. (D) Neurons coding at least one cluster (sub-strategy) of the FSA model with 8 states; cluster left, and/or cluster right, and/or win-stay, lose-switch. (E) Neurons coding at least one current state from *x*_1_(*t*) to *x*_8_(*t*) of the FSA model. (F) Neurons coding at least one next state from *x*_1_(*t*+1) to *x*_8_(*t*+1) of the FSA model.

In our previous study, we detected action-value coding neurons and state-value coding neurons by linear regression analysis, in which action values estimated by the FQ-learning were used as regressors. In this study, we used an augmented regression model (Poisson regression model), including not only variables of the FQ-learning, but also variables of the FSA models. As a result, neurons coding variables of the FQ-learning were still detected (Fig 7A–7C) as in our previous analysis [18], although the performance of the FQ-learning model was worse than that of the FSA model. Significant proportions of neurons in which the firing rates were correlated with action values (*Q*_L_ or/and *Q*_R_) were found in all regions (Fig 7B). Significant proportions of state value- (Fig 7A) and chosen value-coding neurons (Fig 7C) were found mainly in DMS and VS.

A substantial proportion of striatal neurons also coded internal states of the FSA model (Fig 7D–7F). A significant proportion of cluster-coding (*C*_L_, *C*_R_, and/or *C*_WSLS_) neurons were found in VS (Fig 7D), which might be similar to the strategy-coding neurons reported in monkey striatum [26]. The proportion of neurons coding *x*(*t*) in DMS showed a peak during the action execution (Fig 7E). After entry into the left or right hole (and the reward or no-reward tone was presented), populations of *x*(*t*+1) in all regions were increased (Fig 7F), consistent with state transition dependence on reward feedback. Some neurons in DMS showed firing correlated with *x*(*t*+1) even before presentation of the reward or no-reward tone (Fig 7F), which was possible because the reward was highly predictable (90% or 10%) in one of the actions in each block.

Were variables of the FQ-learning and the FSA models separately coded in different neurons? During action execution (500 ms before entry into the L/R hole), neurons coding only the variables of the FQ-learning model (state value, action value, chosen value) were 6.9% (14/204) in DLS, 8.9% (10/112) in DMS, and 2.9% (4/138) in VS. Neurons coding only FSA-related variables (sub strategy, *x*(*t*), *x*(*t*+1)) were 8.8% (18/204) in DLS, 22.3% (25/112) in DMS, and 14.5% (20/138) in VS. Neurons coding both variables were 2.0% (4/204) in DLS, 7.1% (8/112) in DMS, and 8.7% (12/138) in VS. While VS neurons significantly tended to code variables of both models, in DLS and DMS there were no significant tendencies (*p* = 0.10 for DLS, *p* = 0.13 for DMS, and *p* < 0.0001 for VS, chi-squared tests).

**Fig 8:**
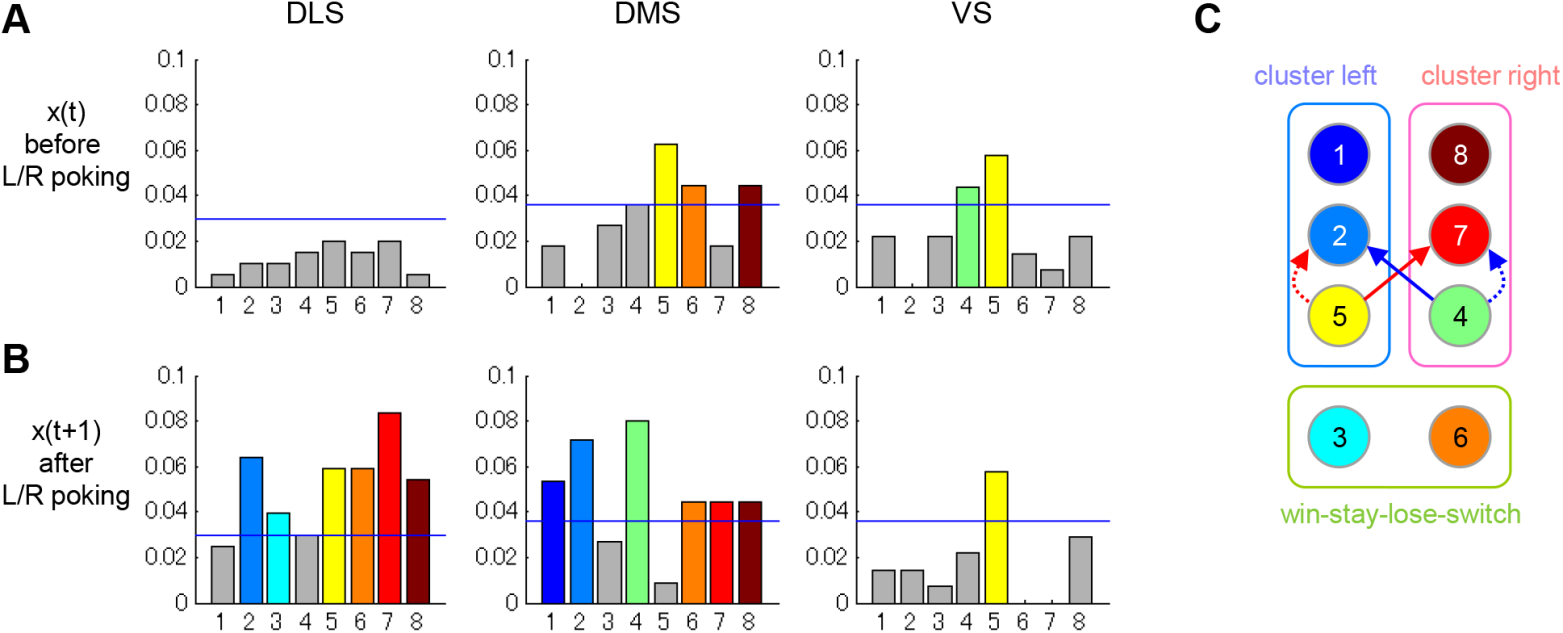
Breakdowns of state-coding neurons shown in Fig 7E and 7F. (A, B), The proportion of neurons coding *x*(*t*) during the 500 ms before entry into the L/R hole, and *x*(*t*+1) during 500 ms after exit from L/R hole, respectively. The color for each state showing a significant proportion (*p* < 0.05, binominal test) corresponds to the color in the simplified diagram of the state transition in the FSA model with 8 states shown in (C). Populations with less than chance probabilities are shown in gray.

Interestingly, not all states were equally coded in the striatum (Fig 8). During action execution (Fig 8A), only the proportion of state-4- and state-5-coding neurons in DMS and VS (also state 6 and 8 in DMS) were statistically significant, and both states preceded an exploratory action in the keep-left and keep-right clusters (Fig 4C). After execution of an action and reward feedback (Fig 8B), representations of most subsequent states appeared in DLS and DMS, while representations of the same state *x*_5_, persisted in VS. Interestingly, states 2 and 7 are major transition targets from states 4 and 5, and these signals, especially, the signal of state 7, were prominent in DLS.

## Discussion

To explore what types of decision-making algorithms are utilized and implemented in the basal ganglia, we evaluated three different strategies for reproducing choice behaviors of rats, and examined their neural correlates in the striatum. We found that the finite state strategy matched the choice behavior of rats most faithfully, both in the normalized likelihood with fitting to the choice sequences and in the statistical properties of the choice behavior in autonomous simulations. Neuronal activity analysis revealed that variables used in both the finite state and value-based strategies were encoded in the striatum. These findings suggest that both finite state and value-based strategies were processed in parallel in brain circuits that include the striatum, while actual choices of rats were predominantly determined by the finite state strategy in the present task.

### Finite state-based strategy

The finite state-based strategy implemented with *N* = 8 states showed a significantly higher prediction accuracy (average likelihood) for rat choice behaviors than the best reinforcement learning model, the FQ-learning model [5] [18]. Furthermore, we compared statistical features of the time course of learning (the number of trials to reach 80% optimality) and the probabilities of repeating the same action after rewarded or non-rewarded outcomes of the rats and the algorithms when faced the same task (Fig 5). We found that only the FSA model with 8 states could reproduce those features similar to the rats. Therefore the FSA model is the best model to predict rat actions in individual trials and also to reproduce generic features of the time course of learning, although we cannot deny the possibility that there might be an even better model in both respects.

The FSA model is conceptually different from the other models. The Q-learning (FQ-learning) models and the ESE models are normative models that prescribe behaviors for maximization of rewards, whereas the FSA model is a descriptive model that seeks only to describe the behavior as it appears in the data [27]. The reformulated Baum-Welch algorithm was used not to find the parameters with which the models maximize the reward, but to find the parameters with which the models mimic the choice behavior of rats. The FSA models do not explain why and how the rats learned the procedure (Fig 4C). If an FSA-like algorithm is implemented in the brain, how could the algorithm learn the appropriate choice and transition probabilities to efficiently obtain a reward? A possible scenario is that rats use the value-based strategy in the beginning of the training. Meanwhile, the finite state strategy monitored behavior to form a procedure that mimicked the value-based strategy without explicit value evaluation. After massive training, the procedure was formed, and the finite state strategy overrode action selection. We speculate that the finite state strategy could be regarded as generalized habit formation. Traditionally, habitual actions are considered automatic responses controlled by simple stimulus-response associations without any associative links to the outcome of those actions [28]. The finite state strategy could be considered as an extended habitual action that depends not only on stimuli, but also internal states. To test this idea, further behavioral experiment will be required.

Internal states of the FSA model were represented in the all three subregions of the striatum (Fig 7E and 7F), while it has been reported that habitual actions involve DLS [28–31]. We speculate that retention of internal states required for the FSA model involves the working memory functions of the prefrontal cortex [32], which can explain the internal state representation in not only DLS, but also DMS and VS, where the prefrontal cortex projects [33].

Analysis of neuronal activities suggests that all striatal areas we recorded, namely, DLS, DMS, and VS, are involved in the finite state strategy. Interestingly, not all states were equally coded in the striatum (Fig 8A and 8B). While codings of *x*_4_(*t*) and *x*_5_(*t*) were found in DMS and VS, coding of *x*_1_(*t*), *x*_2_(*t*), *x*_3_(*t*), and *x*_7_(*t*) was not observed in any areas. Note that *x*_4_(*t*) and *x*_5_(*t*) are the states in which an action is likely to be switched after repeated unrewarded actions at *x*_1_(*t*), *x*_2_(*t*), *x*_8_(*t*) or *x*_7_(*t*). This uneven representation of states suggests that the finite state strategy is implemented in a larger brain circuit that includes the striatum. The requirement of working memory to store the current state suggests the involvement of other brain regions, such as the prefrontal cortex and the hippocampus. Then why are *x*_4_(*t*) and *x*_5_(*t*) are selectively coded in the striatum? It has been reported that the anterior cingulate cortex (ACC) plays an important role in switching behavior evoked by error feedback [34]. The connection from the ACC to the striatum for the execution of switching [35] may be the source of strong coding of *x*_4_(*t*) and *x*_5_(*t*) observed in DMS and VS.

### Value-based strategy

Previous studies have reported that action-value signals are represented in the striatum of rodents [5,7,8], monkeys [4,11,23,36] and humans [12], suggesting that the value-based strategy is implemented in the basal ganglia. Consistent with these reports, our previous study [18] reported that state value signals were most strongly represented in VS, and that action value signals were most strongly represented in DMS during action execution.

In the present study, we reanalyzed the same dataset as the previous study, with a more complex regression model, including not only action values, but also state values, the chosen value, and variables of the FSA model that best explained animal behaviors. We applied lasso regularization to the augmented regression model, and similar results were reproduced; strong state-value coding in VS (Fig 7A), and a peak of the proportion of action-value coding neurons in DMS during action execution (Fig 7B). In addition, we found that the signal of the chosen value, previously reported in monkeys [23,26] and rats [7], was represented in VS in our dataset (Fig 7C).

It has been proposed that DMS is involved in goal-directed actions [28,30] based on lesion studies [37,38]. Formation of goal-directed action is thought to require an association between actions and outcomes, which is analogous to the action value in reinforcement learning. Accordingly, action-value coding in DMS matches the proposal of goal-directed action in DMS. The action value for the selected action, called the chosen value [7,23], which is necessary for updating action values, was observed in VS. Furthermore, consistent with previous reports in rodents [5], state-value representation was observed in VS (Fig 7E). These findings suggest that the value-based strategy is implemented in the striatum, although the final action choices are better characterized by the finite state-based strategy.

### Environmental model-based strategy

The likelihood of the ESE model for the model-based strategy was much lower than that of the FQ-learning model for the value-based strategy or that of the FSA model for the finite state strategy. Thus, rats may not have estimated the reward setting in our task. In this task, four pairs of reward probabilities were used, but in the previous report in human subjects [13], only two pairs were used. Therefore, it might be too difficult for rats to estimate one reward setting from four possible pairs.

### Hierarchical structure in the striatum

The present results support the notion of a hierarchical structure in the cortico-basal ganglia loops, but suggest specific roles for different loops in implementation of the value-based and finite state-based strategies. Representation of state values and sub-strategies (clusters) in VS (Fig 7A and 7D) suggests a role for this region in higher-level decisions, namely, selection of sub-strategies depending on the frequency of reward [39,40]. Robust coding of action values and states responsible for action switching in DMS (Fig 7D and 7G) points to a role for this region in flexible action adaptation. Action coding in DLS was equal to or stronger than that in DMS before movement onset [18], suggesting a major role for this region in action preparation and initiation.

## Materials and Methods

### Ethics statement

All experimental procedures were performed in accordance with guidelines approved by the Okinawa Institute of Science and Technology Experimental Animal Committee.

### Dataset

A part of the dataset used in our previous study [18] was reused in this study. Behavioral and neuronal data were gathered from seven Long-Evans rats. The number of sessions completed by each rat was from 24 to 33. The average (+ standard deviation) of the trials per session was 41.10 (+ 27.58) trials. Neurons stably recorded from at least two sessions were 260 in DLS, 178 in DMS, and 179 in VS (on average, recorded from 2.7 sessions). From this dataset, phasically active neurons (PANs; 204 from DLS, 112 from DMS, and 138 from VS) were extracted based on inter-spike interval statistics. The proportion of inter-spike intervals (ISIs) that was > 1 s of total recoding time (Pr*op*_*ISIs*>1*s*_) was calculated for each neuron [41]. Then, neurons for which Pr*op*_*ISIs*>1*s*_> 0.4 were regarded as PANs.

### Event-aligned spike histograms (EASHs)

Intervals of the six task events (entry into the center hole, onset of the cue tone, offset of the cue tone, exit from the center hole, entry into the left or right hole, and exit from the left or right hole) varied by trials. To align event timings for all trials, event-aligned spike histograms (EASHs) were proposed by Ito and Doya [18]. First, the average duration for each event interval was calculated. Then, spike timings in a certain event interval for each trial were linearly transformed into corresponding averaged event intervals. Finally, histograms of the number of spikes for each 100 ms time window were calculated (Fig 6A, 6D and 6G).

### Decision-making models

Any decision-making models for a single stimulus (state) and binary choice (action) can be defined by the conditional probability of a current action given past experiences:

$$P_{L}(t)=P\left(a(t)=L|e(1:t-1)\right)$$ (2)

where *e*(1:*t*-1) is a simple description of *e*(1), *e*(2),…, *e*(*t*-1). *e*(*t*) is a set of an action and a reward *e*(*t*) = {*a*(*t*), *r*(*t*)}, and action *a*(*t*) and reward *r*(*t*) can be *L* or *R* and 1 or 0, respectively. Behavioral data are composed of a set of sequences (sessions) of actions and rewards. If necessary, we use the index *l* as the index of sessions, for example *a*{*l*}(*t*). The number of trials for session *l* is represented by *T*_*l*_, and the number of sessions is *L*.

To fit parameters to choice data and to evaluate the models, we used the likelihood criterion, which is the probability that the observed data were produced by the model. The likelihood can be normalized, so that it equals 0.5 when predictions are made with chance-level accuracy (*P*_*L*_(*t*) = 0.5 for all *t*). The normalized likelihood is defined by

$$Z={\left[\prod_{l=1}^{L}\left[\prod_{t=1}^{T_{l}}z\{l\}(t)\right]\right]}^{\frac{1}{\sum_{l=1}^{L}T_{l}}}$$ (3)

where *z*{*l*}(*t*) is the likelihood for a single trial:

$$z\{l\}(t)=\{\begin{array}{ccc} P_{L}(t) & \text{if} & a\{l\}(t)=L\\ 1-P_{L}(t) & \text{if} & a\{l\}(t)=R \end{array}.$$ (4)

The (normalized) likelihood can be regarded as the prediction accuracy, namely, how accurately the model predicts actions using past experiences. Generally, models that have a larger number of free parameters can fit data more accurately and thus show a higher likelihood. However, these models may not be able to fit new data due to over-fitting. For fair comparison of models, choice data were divided into training data (101 sessions) and test data (101 sessions). Free parameters of a model were determined to maximize the likelihood of training data. Then, the model was evaluated by the likelihood or the normalized likelihood of the test data (holdout validation). Therefore, in this model fitting, each model was fitted to all training set trials from all seven rats with the same free parameters. Fig 2A represents the normalized likelihood for the total of test 101 sessions. For statistical tests of the normalized likelihood between the models (Fig 2A), we compared the normalized likelihood of each session for the same parameters between the models by a paired-sample Wilcoxon test.

From the above process, we obtained the likelihood of each trial (4) in all sessions (both training and test data) for each model with the parameters estimated by training data. To compare fitting performance, we averaged the sequences of the likelihoods of the last 20 trials over all blocks with higher or lower reward probabilities (Fig 2B and 2C). To test significant differences between the FSA model and the DFQ model, the Mann-Whitney U test was applied to the likelihoods for every trial.

Note that the normalized likelihood depends on the number of trials. If an animal’s choice probability does not change over trials, namely, *P*(*a*(*t*) *=* L) *= P*, and model prediction *P*_*L*_(*t*) is also constant *P*_*L*_, then the expected normalized likelihood for *T* trials is given by

$$\hat{Z}(T)=\sum_{t=0}^{T}\left(\begin{array}{c} T\\ t \end{array}\right)P^{t}{\left(1-P\right)}^{T-t}{\left(P_{L}{}^{t}{\left(1-P_{L}\right)}^{T-t}\right)}^{1/T}.$$ (5)

This expected normalized likelihood rapidly decreases when the number of trials increases, and when *T* goes to infinite, it converges to

$$\hat{Z}(\infty )=P_{L}{}^{P}{\left(1-P_{L}\right)}^{\left(1-P\right)}.$$ (6)

For example, let’s assume that a rat’s choice probability is *P* = 0.8 and model A predicts it perfectly by *P*_*L*_ = 0.8, the (normalized) likelihood is $\hat{Z}(1)=0.68$, it’s less than *P*_*L*_, and it decreases to $\hat{Z}(\infty )=0.61$ when *T* increases. If model B predicts with *P*_*L*_ = 0.7, $\hat{Z}(1)=0.62$ and $\hat{Z}(\infty )=0.59$, the difference in the normalized likelihood between model A and model B also decreases (0.07 → 0.02) when *T* changes from 1 to infinity. This is the reason why the normalized likelihoods of models shown in Fig 2A (*T* = 16856 trials) are much less than the likelihoods shown in Fig 2B and 2C (*T* = 1 trial).

### Markov models

*d*th-order Markov models are the simplest non-parametric models. They predict an action at trial *t*, *a*(*t*), from the past *d*-length sequence of experiences before *t*, *e*(*t-d*:*t*-1). The prediction of the *d*th-order Markov model was given by the following:

$$P_{L}(t)=\frac{N_{L}\left(e(t-d:t-1)\right)+1}{N_{L}\left(e(t-d:t-1)\right)+N_{R}\left(e(t-d:t-1)\right)+2}$$ (7)

where *N*_*i*_(*e*(*t* − *d*:*t* − 1)) is the number of *i* (L or R) chosen after every *d*-length sequence of the exact same sequence as *e*(*t-d*:*t*-1) in the whole training data [5]. The *d*th-order Markov model has more than 4^*d*^ free parameters because there are four types of possible experiences in a single trial (more precisely, the number of the parameters is 4^*d*^+4^(*d*−1)^+⋯+4. The *d*th-order Markov model uses the 1st-order Markov model for the prediction of the first trial in a session, and 2nd-order Markov model for the second trial). The Markov models are purely descriptive models, but they provide a useful measure to objectively evaluate other models.

### Q-learning models

The DFQ-learning model [5,18], which is an extension of the Q-learning model and which includes the original Q-learning model with certain parameters, is useful to test the Q-learning family. A key component of the DFQ-learning (and Q-learning) model is to use action values (*Q*_*L*_ and *Q*_*R*_) as predictions of the future cumulative reward that the agent would obtain after selecting left or right, respectively. The model selects an action that has a higher action value with a higher probability:

$$P_{L}(t)=\frac{1}{1+\exp\left\{-\left(Q_{L}(t)-Q_{R}(t)\right)\right\}}.$$ (8)

After determining the reward outcome, action values are updated by:

$$Q_{i}(t)=\{\begin{array}{ccc} (1-\alpha_{1})Q_{i}(t-1)+\alpha_{1}\kappa_{1} & \text{if} & a(t-1)=i,r(t-1)=1\\ (1-\alpha_{1})Q_{i}(t-1)-\alpha_{1}\kappa_{2} & \text{if} & a(t-1)=i,r(t-1)=0\\ \left(1-\alpha_{2})Q_{i}(t-1\right) & \text{if} & a(t-1)\neq i,r(t-1)=1\\ \left(1-\alpha_{2})Q_{i}(t-1\right) & \text{if} & a(t-1)\neq i,r(t-1)=0 \end{array}$$ (9)

where *i* ∈ {*L*,*R*}, *α*_1_ is the learning rate for the selected action, *α*_2_ is the forgetting rate for the action not chosen, *κ*_1_ represents the strength of reinforcement by reward, and *κ*_2_ represents the strength of the aversion resulting from the non-reward outcome. This set of equations can be reduced to the standard Q-learning by setting *α*_2_ = 0 (no forgetting for actions not chosen) and *κ*_2_ = 0 (no aversion from a lack of reward). The FQ-model is a version introducing the restriction *α*_1_ = *α*_2_.

For the Q-learning models, we considered cases of fixed parameters and time-varying parameters. For fixed parameter models, *α*_1_, *α*_2_, *κ*_1_, and *κ*_2_ are free parameters. For time-varying parameters, *α*_1_, *α*_2_, *κ*_1_, and *κ*_2_ are not free parameters; they are assumed to vary according to the following:

$$\begin{array}{ccc} \alpha_{j}(t)=\alpha_{j}(t-1)+\varsigma_{j} & \text{for} & j\in \{1,2\}\\ \kappa_{j}(t)=\kappa_{j}(t-1)+\xi_{j} & \text{for} & j\in \{1,2\} \end{array}$$ (10)

where *ζ*_*j*_ and *ξ*_*j*_ are noise terms drawn independently from the Gaussian distribution *N*(0,*σ*_*α*_^2^) and *N*(0,*σ*_*κ*_^2^), respectively. *σ*_*α*_ and *σ*_*κ*_ are free parameters that control the magnitude of the change. The predictive distribution *P*(*h*(*t*)| *e*(1:*t*-1)) of parameters *h* = [*Q*_*L*_, *Q*_*R*_, *α*_1_, *α*_2_, *κ*_1_, *κ*_2_] given past experiences *e*(1:*t*-1) was estimated using the particle filter [4,5]. The action probability *P*_*L*_(*t*) was obtained from Eq (8) with the mean of the predictive distribution of *Q*_*L*_ (*t*) and *Q*_*R*_ (*t*). In this study, 5,000 particles were used for the estimation.

### Environmental state estimation (ESE) models

The ESE model estimates a hidden environmental state, namely, the reward setting from past experience, using the knowledge that reward probabilities should be one of the following: (90, 50%), (50, 10%), (50, 90%) and (10, 50%) (five trials with zero reward probability inserted in the middle of each session were not considered.). The ESE model also assumes that the reward setting is changed with a small probability *ε* for each trial:

$$P\left(s(t)|s(t-1)\right)=\{\begin{array}{ccc} 1-\varepsilon & \text{if} & s(t)=s(t-1)\\ \varepsilon /3 & \text{if} & s(t)\neq s(t-1) \end{array}$$ (11)

where *s*(*t*) ∈ {1,2,3,4} is the index of reward setting at trial *t* corresponding to (90, 50%), (50, 10%), (50, 90%) and (10, 50%), respectively. The prediction of the reward setting at trial *t* for all *s*(*t*) is obtained using

$$P\left(s(t)|e(1:t-1)\right)=\sum_{s(t-1)=1}^{4}P\left(s(t)|s(t-1)\right)P\left(s(t-1)|e(1:t-1)\right)$$ (12)

where *P*(*s*(*t*-1)| *e*(1:*t*-1)) is the prior probability of the reward setting. The prior probability for *t* = 1 was set to 1/4 for each *s*. Based on this prediction, action values are given by

$$Q_{i}(t)=\kappa \sum_{s(t)=1}^{4}P\left(r(t)=1|s(t),a(t)=i\right)P\left(s(t)|e(1:t-1)\right)$$ (13)

where *P*(*r*(*t*) = 1| *s*(*t*), *a*(*t*) = *i*) is the reward probability for the reward setting *s*(*t*) and action *i*. *κ* is the magnitude of the reward. An actual action, *a*(*t*), is selected according to the action probability, which is calculated from Eq (8) with the action values. After knowing the reward outcome, *r*(*t*), the posterior probability of the reward setting for all *s*(*t*), was updated using Bayes’ theorem:

P(s(t)|e(1:t))∝P(a(t),r(t)|s(t),e(1:t−1))P(s(t)|e(1:t−1)). (14)

The first factor of the right side can be decomposed to

P(a(t),r(t)|s(t),e(1:t−1))=P(r(t)|a(t),s(t),e(1:t−1))P(a(t)|s(t),e(1:t−1)) (15)

where the first factor on the right side of this equation can be simply written as *P*(*r*(*t*) | *a*(*t*),*s*(*t*)) because this factor comes from the reward probability setting of the task and is assumed to be independent of the past experience of rats, *e*(1:*t*-1). The second factor is the action probability of the agent. Although the agent estimates the current reward setting, *s*(*t*), from past experience, *e*(1:*t*-1), the agent cannot directly observe *s*(*t*). In other words, the action probability should be the same for the same past experience, *e*(1:*t*-1), without being affected by the true hidden state, *s*(*t*). Therefore, the second factor can be ignored because it takes the same values for all *s*(*t*). Then, Eq (14) is simplified to

P(s(t)|e(1:t))∝P(r(t)|s(t),a(t))P(s(t)|e(1:t−1)). (16)

Similar to the Q-learning models, we considered the cases of fixed and time-varying parameters. For fixed parameter models, *ε* and *κ* are free parameters. For time-varying parameters, *ε* and *κ* were assumed to vary by a random walk with the Gaussian distribution *N*(0, *σ*_*ε*_^2^) and *N*(0, *σ*_*κ*_^2^), respectively. *σ*_*ε*_ and *σ*_*κ*_ are the free parameters that control the magnitude of the change.

### Finite state agent (FSA) models

FSA models are non-parametric models that have internal variables *x* taking *N* possible states, *x* ∈ {1,2,⋯,*N*}. The initial distribution of the state is described by

$$q^{n}=P\left(x(t=1)=n\right).$$ (17)

The probability of an action selection depends on the state and is defined by

$$\pi_{n}\left(a(t)\right)=P\left(a(t)|x(t)=n\right).$$ (18)

After execution of an action and the subsequent reward outcome, the state is probabilistically moved to another state according to the state transient function:

$$U_{n}^{m}\left(a(t),r(t)\right)=P\left(x(t+1)=m|x(t)=n,a(t),r(t)\right).$$ (19)

*q* ^*n*^, *π*_*n*_(*a*), $U_{n}^{m}(a,r)$, and *N* are the free parameters of the FSA models. Considering the probabilistic constraints, $\sum_{n=1}^{N}q^{n}=1$, $\sum_{a=\{L,R\}}^{}\pi_{n}(a)=1$, $\sum_{m=1}^{N}U_{n}^{m}(a,r)=1$, and symmetric constraints *q* ^*l*^ = *q* ^*N*−*l*+1^, $\pi_{l}(a)=\pi_{N-l+1}(\bar{a})$, $U_{l}^{l'}(a,r)=U_{N-l+1}^{N-l'+1}(\bar{a},r)$, for *l*, *l’* = 1, 2,…, *N*, where $\bar{a}$ is the other of actions, and the number of free parameters is (*N*/2-1) + *N*/2 + 2*N*(*N*-1) = 2*N* ^2^-*N-*1 when *N* is an even number and (*N*-1)/2 + (*N*-1)/2 + 2(*N*-1)^2^ = 2*N* ^2^ - 3*N* +1 when *N* is an odd number.

The FSA model can be regarded as an extended version of the hidden Markov model (HMM). However, unlike the HMM, in the FSA model, the state transition probability depends on the action and reward. In the HMM, the Baum-Welch algorithm [19], a form of the EM algorithm, is used to find the parameters that maximize the likelihood of the given data. We reformulated the Baum-Welch algorithm for the FSA model.

### EM algorithm for FSA model

1. Initialize parameters *q* ^*n*^, *π*_*n*_(*a*), $U_{n}^{m}(a,r)$, so the probabilistic constraints, $\sum_{n=1}^{N}q^{n}=1$, $\sum_{a=\{L,R\}}^{}\pi_{n}(a)=1$, $\sum_{m=1}^{N}U_{n}^{m}(a,r)=1$, and the symmetric constraints, *q* ^*l*^ = *q* ^*N*−*l*+1^, $\pi_{l}(a)=\pi_{N-l+1}(\bar{a})$, $U_{l}^{l'}(a,r)=U_{N-l+1}^{N-l'+1}(\bar{a},r)$, are satisfied (*N* is a fixed parameter). In this study, we set *q* ^*n*^ = 1/*N* for all *n*, *π*_*n*_(*L*) = 0.9−0.8(*n*−1)/(*N*−1) and *π*_*n*_(*R*) = 1−*π*_*n*_(*L*) for all *n*, and $U_{n}^{m}(a,r)=1/N$ for all *n*, *m*, *a*, and *r*.

2. E-step

Estimate the posterior probability of the state for all *t* and *l*, *γ* ^*n*^{*l*}(*t*) = *P*(*x*{*l*}(*t*) = *n*|*a*{*l*}(1:*T*_*l*_),*r*{*l*}(1:*T*_*l*_)), assuming that the data were produced with current parameters.

First, estimate *α* ^*n*^{*l*}(*t*) = *P*(*x*{*l*}(*t*) = *n*|*a*{*l*}(1:*t*),*r*{*l*}(1:*t*)), the posterior probability of a state at trial *t* given the data from 1 to the current trial *t*. The probability can be obtained iteratively from *t* = 1 to *t* by:

$$\alpha^{n}\{l\}(t)=\frac{\pi_{n}\left(a\{l\}(t)\right)\sum_{m}U_{m}^{n}\left(a\{l\}(t-1),r\{l\}(t-1)\right)\alpha^{m}\{l\}(t-1)}{\sum_{n'}\pi_{n'}\left(a\{l\}(t)\right)\sum_{m'}U_{m'}^{n'}\left(a\{l\}(t-1),r\{l\}(t-1)\right)\alpha^{m'}\{l\}(t-1)},$$ (20)

where *α* ^*n*^{*l*}(*t* = 1) = *q* ^*n*^.

Next, estimate *γ* ^*n*^{*l*}(*t*) = *P*(*x*{*l*}(*t*) = *n*|*a*{*l*}(1:*T*_*l*_),*r*{*l*}(1:*T*_*l*_)), the posterior probability of a state, given all data using the already obtained *α* ^*n*^{*l*}(*t*) and an additional variable, *χ* ^*nm*^{*l*}(*t*) = *P*(*x*{*l*}(*t*) = *n*, *x*{*l*}(*t* + 1) = *m* | *a*{*l*}(1:*T*_*l*_),*r*{*l*}(1:*T*_*l*_)). *γ* ^*n*^{*l*}(*t*) and *χ* ^*nm*^{*l*}(*t*) are obtained in a backward manner from *t = T*_*l*_ (or *T*_*l*_ *-*1 for *χ* ^*nm*^) to 1 in parallel. For *t* = *T*_*l*_, *γ* ^*n*^{*l*}(*T*_*l*_) = *α* ^*n*^{*l*}(*T*_*l*_). Then, *χ* ^*nm*^{*l*}(*T*_*l*_−1) is obtained using

$$\chi^{nm}\{l\}(t)=\frac{\gamma^{m}\{l\}(t+1)U_{n}^{m}\left(a\{l\}(t),r\{l\}(t)\right)\alpha^{n}\{l\}(t)}{\sum_{n'}U_{n'}^{m}\left(a\{l\}(t),r\{l\}(t)\right)\alpha^{n'}\{l\}(t)},$$ (21)

and *γ* ^*n*^{*l*}(*T*_*l*_−1) is obtained using

$$\gamma^{n}\{l\}(t)=\sum_{m}\chi^{nm}\{l\}(t).$$ (22)

By repeating (21) and (22), *γ* ^*n*^ and *χ* ^*nm*^ are obtained for all trials.

3. M-step

Update model parameters, assuming the state probabilities *γ* ^*n*^ and *χ* ^*nm*^ are true. Given the probabilistic and symmetric constraints, parameters are updated using

$$q^{n}=q^{N-n+1}=\frac{\sum_{l=1}^{L}\left[\gamma^{n}\{l\}(1)+\gamma^{N-n+1}\{l\}(1)\right]}{2L},$$ (23)

$$\pi_{n}(a)=\pi_{N-n-1}(\bar{a})=\frac{\sum_{l=1}^{L}\sum_{t=1}^{T_{l}}\left[\gamma^{n}\{l\}(t)\delta \left(a\{l\}(t)=a\right)+\gamma^{N-n+1}\{l\}(t)\delta \left(a\{l\}(t)=\bar{a}\right)\right]}{\sum_{l'=1}^{L}\sum_{t'=1}^{T_{l'}}\left[\gamma^{n}\{l'\}(t')+\gamma^{N-n+1}\{l'\}(t')\right]},$$ (24)

and

$$\begin{array}{l} U_{n}^{m}(a,r)=U_{N-n+1}^{N-m-1}(\bar{a},r)\\ =\frac{\sum_{l=1}^{L}\sum_{t=1}^{T_{l}-1}\left[\chi^{nm}\{l\}(t)\delta \left(a\{l\}(t)=a,r\{l\}(t)=r\right)+\chi^{N-n+1,N-m+1}\{l\}(t)\delta \left(a\{l\}(t)=\bar{a},r\{l\}(t)=r\right)\right]}{\sum_{l'=1}^{L}\sum_{t'=1}^{T_{l}-1}\left[\gamma^{n}\{l'\}(t')\delta \left(a\{l'\}(t')=a,r\{l'\}(t')=r\right)+\gamma^{N-n+1}\{l'\}(t')\delta \left(a\{l'\}(t')=\bar{a},r\{l'\}(t')=r\right)\right]} \end{array}$$ (25)

4. Check for convergence of the parameters. If the convergence criterion is not satisfied, return to step 2. In this study, we stopped the iteration when the maximum change of the parameters was less than 0.00001.

#### Definition of clusters of states in FSA models

Let us consider a certain FSA model in which a state transition frequently occurs between state 1 and 2, but states 1 and 2 rarely transition to other states. In this case, the set of state 1 and 2 may be regarded as a state of a higher order (a cluster). This structure of states is helpful to understand the meanings of the procedures coded in the FSA models. To find the most plausible clustering structure, we calculated the state transition probability, $T_{n}^{m}=P\left(x(t+1)=m|x(t)=n\right)$, by

$$T_{n}^{m}=\sum_{l=1}^{L}\sum_{t=1}^{T_{l}-1}\left[\chi^{nm}\{l\}(t)/\gamma^{n}\{l\}(t)\right],$$ (26)

where *χ* ^*nm*^{*l*}(*t*) and *γ* ^*n*^{*l*}(*t*) are estimated from the behavioral data. Then, we defined the cluster index by the average of the state transition probabilities in the clusters:

$$\frac{1}{N_{c}}\sum_{i}\sum_{m\in h_{i}}\sum_{n\neq m,n\in h_{i}}T_{n}^{m},$$ (27)

where *i* is an index of the clusters, *h*_*i*_ is a set of states included in the cluster *i*, and *N*_*c*_ is the number of terms in the summations. For instance, if 6 states are clustered into [1 2] and [3 4 5 6] for the FSA model with 6 states, the cluster index is $\frac{1}{14}\left[T_{1}^{2}+T_{2}^{1}+T_{3}^{4}+T_{3}^{5}+T_{3}^{6}+T_{4}^{3}+T_{4}^{5}+T_{4}^{6}+T_{5}^{3}+T_{5}^{4}+T_{5}^{6}+T_{6}^{3}+T_{6}^{4}+T_{6}^{5}\right]$. We calculated the cluster index for all possible ways of clustering for each FSA model with 4, 6, and 8 states, assuming that all clusters include more than one state and that all states belong to any cluster. Then, the clustering showing the highest cluster index was selected as the most plausible clustering structure (Fig 4).

### Comparison of simulated behavior

To test whether the models can generate behavioral data that have the same statistics as behavioral data, we compared the simulated behavior of the models with the behavioral results. First, as a measure of adaptation speed to the change of reward probabilities, we calculated the mean number of trials in one block for the higher reward probability settings, (90, 50%) and (50, 90%), and for the lower reward probability settings, (50, 10%) and (10, 50%), from all 202 recorded sessions. Because each session consists of four blocks with different reward probability settings, there were 404 higher reward blocks and 404 lower reward blocks in the data. Second, as a measure of the strategy utilized by the rats, the probability that the same action was selected after a rewarded or non-rewarded trial, *P*(*a*(*t*+1) = *a*(*t*)| *r*(*t*) = 1) and *P*(*a*(*t*+1) = a(*t*)| *r*(*t*) = 0), for higher and lower reward probability settings, respectively, was calculated. These four action probabilities were calculated from the last 20 trials in four blocks for each session, and then the mean of these probabilities was calculated from all 202 sessions. Third, we conducted a model simulation for 202 sessions in which the same block sequences as those used in all 202 sessions were applied. Then, the six statistics noted above were calculated from the simulated data: [the number of trials in a block, *P*(*a*(*t*+1) = *a*(*t*)| *r*(*t*) = 1), *P*(*a*(*t*+1) = a(*t*)| *r*(*t*) = 0)] x [higher, lower reward probability setting]. By repeating this simulation 10,000 times, the approximate distribution for each statistic was obtained.

Note that the statistics calculated from the rats are random variables. If the hypothesis that the choice behavior of rats was sampled from a certain model is true, then statistics obtained from behavioral measures should fall within the distribution of the statistics (inside the confidence interval with 1—*ε*) calculated by the model. Otherwise, the hypothesis is rejected. We considered six different tests for the same hypothesis, so the chance of at least one false rejection is much higher than *ε*. Therefore, the confidence interval for each statistic was set to 1—*ε*/6, so the chance of at least one false rejection is *ε* (Bonferroni Method). In this study, *ε* was set to 0.05. We tested the Q-, FQ-, and DFQ-learning models in addition to the ESE model in which the parameters were fixed. The FSA models with 4, 6, and 8 states were also tested. The free parameters that maximize the likelihood of the training data were used for the simulation.

The distributions of the statistics in one session (Fig 5A–5C) were calculated from 202 behavioral sessions and 10,000 x 202 sessions for the models. As a result, the shape of the distribution is smoother for the models than for behavioral data.

### Lasso regularization of Poisson regression

Linear regression is a popular method to find regressors that can explain the change in neuronal activity, where spikes are assumed to be sampled from a normal distribution. However, in the precise sense, this assumption is not correct because spikes take only non-negative integers. The lower the firing rate of the neuron is, the bigger the gap from the assumption is.

Therefore, in the present study, we used Poisson regression assuming that the spikes are sampled from a Poisson distribution (a distribution of non-negative integer variables). In Poisson regression, the expected number of spikes at trial *t*, *μ*(*t*), is predicted by the following exponential function,

$$\mu (t)=\exp(\beta_{0}+\beta_{1}x_{1}(t)+\beta_{2}x_{2}(t)+\cdots +\beta_{p}x_{p}(t))$$ (28)

where *x*_*i*_ are regressors and *β*_*i*_ are regression coefficients. The prediction of the number of the spikes at trial *t* is represented by a Poisson distribution with the average *μ*(*t*),

$$\text{Poi}(y|\mu (t))=e^{-\mu (t)}\frac{\mu {\left(t\right)}^{y}}{y!}.$$ (29)

Optimal regression coefficients are determined so that the objective function, namely, the log likelihood for all trials,

$$l(\beta )=\sum_{t=1}^{\text{T}}\text{log}\left(\text{Poi}(y(t)|\mu (t))\right)$$ (30)

is maximized. For this calculation, a function in MATLAB Statistics and Machine Learning Toolbox “glmfit(X, y, ‘poisson’)” is available. To select minimum regressors to explain the spikes among many and redundant regressors is, to add a penalty term for large *β* to the objective function,

$$l(\beta )=\sum_{t=1}^{\text{T}}\text{log}\left(\text{Poi}(y(t)|\mu (t))\right)-\lambda \sum_{j=1}^{p}\left|\beta_{j}\right|$$ (31)

where *λ* is a free parameter called the regularization coefficient, and |*β*_*j*_| is the absolute value of *β*_*j*_ (this method is called *lasso* [24]). It has the property that if *λ* is sufficiently large, some of the coefficients *β* are driven to zero [42]. In the present study, all regressors were normalized, so that the average was 0 and the variance was 1. Then, *λ* was optimized by 5-fold cross-validation for each time bin for each neuron, and regression coefficients were obtained. For these calculations, we used a function in the MATLAB Statistics and Machine Learning Toolbox,“lassoglm(X, y, ‘poission’, ‘cv’, 5)”. The regressors with non-zero coefficients were regarded as candidates for minimum regressors. Then, to calculate a *p*-value indicating the probability that each candidate could be incorrectly selected, we applied Poisson regression (MATLAB function, glmfit) to the regression model including only these candidates as regressors. We selected candidates for minimal regressors that had *p*-values < 0.01.

Proportions of neurons coding variables shown in Figs 7 and 8 are the fraction of neurons for which corresponding variables were regarded as minimal regressors for each time bin. The significance of the proportion (*p* < 0.05) was calculated with a binomial test, assuming that the probability that a regressor could be selected incorrectly was *p* = 0.01.
